# Importance of genetic testing in childhood cancer survivors for hereditary cancer predisposition syndromes

**DOI:** 10.1186/s13053-026-00341-2

**Published:** 2026-04-15

**Authors:** Anja Urbas, Polona Ušaj, Boštjan Šeruga, Mateja Krajc, Simona Hotujec, Vida Stegel, Lorna Zadravec Zaletel

**Affiliations:** 1 Faculty of Medicine, University of Ljubljana, Vrazov trg 2, Ljubljana, 1000 Slovenia; 2 Division of Medical Oncology, Institute of Oncology Ljubljana, Zaloška cesta 2, Ljubljana, 1000 Slovenia; 3 Department of Clinical Cancer Genetics, Institute of Oncology Ljubljana, Zaloška cesta 2, Ljubljana, 1000 Slovenia; 4 Department of Molecular Diagnostics, Institute of Oncology Ljubljana, Zaloška cesta 2, Ljubljana, 1000 Slovenia; 5 Biotechnical Faculty, University of Ljubljana, Jamnikarjeva 101, Ljubljana, 1000 Slovenia; 6 Division of Radiotherapy, Institute of Oncology Ljubljana, Zaloška cesta 2, Ljubljana, 1000 Slovenia

**Keywords:** Pediatric cancer, Hereditary cancer predisposition syndrome, Genetic predisposition to cancer, Genetic testing, Pathogenic/likely pathogenic variant, Second primary tumor, Cascade genetic testing

## Abstract

**Background:**

Around 10% of children with cancer carry a germline pathogenic or likely pathogenic variant (P/LPV) in one of the cancer predisposition genes. The objective of this study was to evaluate the frequency of genetic testing for hereditary cancer predisposition syndromes (HCPSs) among adults treated for childhood cancer in Slovenia and to characterize the P/LPVs identified in those who opted for genetic testing. We also aimed to analyse how a positive genetic test influences cascade testing in blood relatives.

**Methods:**

We included patients born between 1970 and 2000 who were diagnosed and treated for cancer between ages 0 − 18 into this retrospective, descriptive, cross-sectional study. Data were collected from the Cancer Registry of the Republic of Slovenia, Long-term Follow-Up Clinic (LTFUC) and the Department of Clinical Cancer Genetics (DCCG) databases. The proportion of tested individuals was calculated with a 95% confidence interval (CI) using the Wald’s method.

**Results:**

Of 1,475patients diagnosed and treated for cancer in childhood, 59 (4.0%; 95% CI: 3.0–5.0%) underwent genetic testing, with 35.6% (21/59) testing positive. The P/LPV were most frequently found in *RB1*, *NF1*, *RET* and *BRCA2* genes. In 61.9% (13/21) of positive cases, an identified cancer predisposition syndrome was linked to the childhood cancer; in 95.2% (20/21) it conferred an increased adult cancer risk. Among six patients with P/LPV and second primary cancer (SPC), two had SPC with possible association with the HCPS. Cascade genetic testing was performed in 38.1% (8/21) of positive families and was documented for 35 relatives of 8 carriers of P/LPV. Overall, 60% (21/35) of relatives were positive at genetic testing and 47.6% (10/21) of developed cancer. In 90% (9/10) of relatives the cancer could be linked to the HCPS.

**Conclusions:**

Only 4% of childhood cancer survivors underwent genetic testing for HCPS. Since hereditary cancer predisposition identified in childhood may also increase cancer risk in adulthood, universal cancer genetic counselling and testing should be considered in childhood to timely enable preventive options for early cancer detection and risk reduction procedures in carriers of P/LPV. In parallel, cascade genetic testing among blood relatives may also offer preventive opportunities to those at risk.

## Introduction

According to the World Health Organization (WHO) there are around 400,000 cases of childhood cancer per year around the world [1, 2]. Among pediatric patients, the most frequently diagnosed malignancies are leukemia, brain tumors, lymphomas, neuroblastoma, and Wilms’ tumor. In EU countries, the estimated five-year survival rates vary considerably between individual nations. Based on the five-year survival rates of 84% for the period 2010–2014, Slovenia ranks among the EU countries with better survival outcomes [3].

Pediatric cancer differs from adult cancer in etiology, treatment, and prognosis. The causes of cancer in children remain poorly understood [3]. Until recently, the prevalence of pediatric cancer attributable to genetic predisposition was generally considered to be very low. However, recent reports indicate that around 10% of children with cancer carry a germline pathogenic or likely pathogenic variant (P/LPV) in a cancer predisposition gene (CPG) [4].

Studying patients with hereditary cancer predisposition syndromes (HCPSs) plays an important role in enhancing preventive approaches, including cancer prevention strategies, early screening and detection, personalized treatment plans, ongoing monitoring, and psychological support for both patients and their families [5].

Several clinical features may raise suspicion of a HCPS in children: a family history of the same or related cancers, bilateral or multifocal tumors, unusually early onset compared to sporadic cases, physical features consistent with a specific predisposition syndrome, or tumor types commonly linked to inherited risk [4, 6]. Children with these characteristics should be referred for genetic counselling.

In Slovenia, systematic genetic testing for HCPSs in children diagnosed with cancer has only recently begun. It remains unclear how many individuals diagnosed with cancer during childhood or adolescence have ever undergone cancer genetic counselling and testing later in life or adulthood. Identifying HCPSs early in life could potentially prevent the development of a second primary cancers (SPCs) in adulthood or enable their earlier detection and in parallel, cascade genetic testing among blood relatives may also offer preventive opportunities to those at risk.

Our aim was to analyse: (i) what proportion of adults who are childhood and adolescence cancer survivors (hereafter childhood survivors) were tested for hereditary cancer predisposition and (ii) whether these hereditary predispositions were associated with adult-onset cancers. We also assessed cascade genetic testing in families identified with hereditary cancer predisposition.

## Methods

### Data sources and analytical cohort of patients

**Fig. 1:**
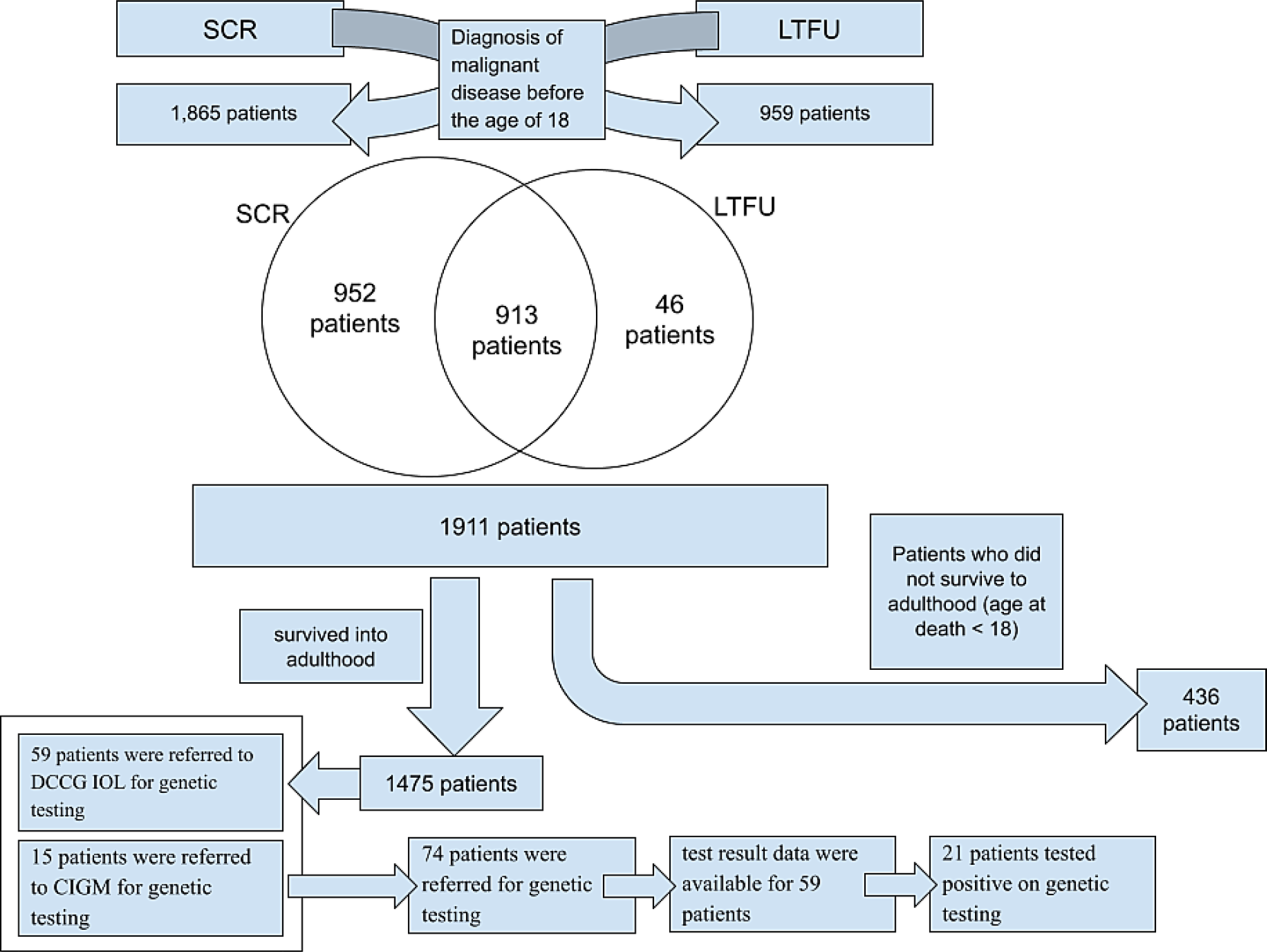
Schematic representation of included and genetically assessed patients. Legend: SCR – Slovenian Cancer Registry, LTFUC – Long-term Follow-up Clinic, DCCG – Department of Clinical Cancer Genetics, IOL – Institute of Oncology Ljubljana, CIGM – Clinical Institute of Genomic Medicine

Data were first obtained from the Slovenian Cancer Registry (SCR), that contains data of all cancer diagnoses since 1950, when compulsory reporting of cancer diagnosis started in Slovenia, and from the national database of the Long-Term Follow-Up Clinic (LTFUC) that was established in 1986 (Fig. 1). LTFUC offers a life-long long-term follow-up to all Slovenian childhood cancer survivors and conducts a population-based studies in the field of late sequelae.

Our analytical cohort included patients born between 1970 and 2000 who had been diagnosed and treated for cancer during childhood and/or adolescence, defined in this study as ages 0–18 years. The upper age limit was set at 18 years, corresponding to the age of majority in Slovenia. While international guidelines commonly define childhood and adolescent cancers as those diagnosed up to and including 19 years of age [7], we applied a more restrictive age cutoff. The cohort was subsequently linked with the database of the Department of Clinical Cancer Genetics (DCCG) at the Institute of Oncology Ljubljana (IOL) to identify adult survivors of childhood and adolescent cancer who had been referred for genetic testing (Fig. 1). Genetic testing for hereditary cancer predisposition has been available at the IOL since 1999, and the DCCG serves as the national reference centre for referrals when a cancer predisposition syndrome is suspected.

In the SCR, we identified 1,865 patients who met our inclusion criteria. In the LTFUC database, we identified 959 eligible patients. Noting that many patients had died before reaching adulthood, we excluded 436 patients who did not survive to adulthood (age at death < 18). The overlap between these two data sources comprised 913 patients. Consequently, our analytical cohort consisted of 1,911 patients in total (taking into account 913 patients present in both datasets). After excluding those who did not survive to adulthood, 1,475 adult survivors remained in the final analytical cohort, as shown in Fig. 1.Among the 913 patients present in both LTFUC and SCR, 54 had also been referred to DCCG, IOL for genetic counselling and testing. In addition, we identified one patient recorded in LTFUC but not in SCR, and four patients listed in SCR but not in LTFUC, who had also been referred for testing. Altogether, we identified 59 patients referred to DCCG, IOL for genetic testing. Furthermore, LTFUC records provided information on 15 additional patients with malignant disease listed in SCR who had not been referred to DCCG, IOL but to the Clinical Institute of Genomic Medicine (CIGM) at the University Clinical Centre Ljubljana. In total, 74 patients from our analytical cohort were referred for genetic assessment (Fig. 1). Not all referred patients underwent genetic testing.

Using data from the SCR, DCCG database, LTFUC medical records OIL, we conducted a detailed analysis of the patients who had undergone genetic testing. We reviewed their childhood cancer diagnoses and age at diagnosis, and calculated interval between the initial cancer diagnosis and referral for genetic testing. For patients with a positive test result, we determined the type of genetic test performed (screening, targeted testing, incidental finding, or confirmation of a variant detected in tumor), the gene, and the type of mutation. We also examined whether the primary cancer and any SPC were associated with a confirmed hereditary cancer syndrome.

Using the data from DCCG, we also obtained information on relatives of patients with positive test who had undergone genetic testing at IOL. We checked whether any family members had developed cancers that are nowadays known to be part of a certain HPCS. All cancer diagnoses were classified according to the International Statistical Classification of Diseases and Related Health Problems, 10th Revision (ICD-10), issued by the WHO. We accessed ICD-10 via https://mediately.co/si/icd (accessed July 2025).

### Statistical analysis

Data were collected from January 2025 to June 2025 and subsequently analysed from June 2025 to August 2025. The initial analysis included descriptive statistics of demographic, clinical, and genetic characteristics. For numerical variables, we reported means with corresponding standard deviations, and medians with interquartile ranges (IQR). For the proportion of patients referred to oncogenetic counseling, we calculated 95% confidence intervals (CI) using the Wald’s method. Non-parametric Mann–Whitney U-test was applied to compare the age at diagnosis of the primary cancer and SPC between patients with positive and negative genetic test.

Pseudo-anonymized data were statistically analyzed using Jamovi software [8]. Two-sided p-values below 0.05 were deemed statistically significant. No adjustment for multiple testing was performed.

### Ethical approval

Prior to study initiation, approvals of the National Medical Ethics Committee of the Republic of Slovenia (application no. 0120–576/2024–2711), Ethics Committee of the IOL (application no. ERIDEK-0088/2024) and the Committee for Scientific Review of Research Protocols (application no. ERID-KSOPR-0075/2024) were obtained.

## Results

### Proportion of adults referred for genetic testing

Among 1,911 childhood cancer patients identified in our analytical cohort 74 (3.9%, 95% CI: 3.0–4.7%) individuals were referred for genetic counselling and testing, primarily based on clinical suspicion. After excluding those who had died before adulthood, these 74 patients represented 5.0% (95% CI: 3.9–6.1%) of all surviving adults that were treated for cancers in the childhood. Of those referred, 59 (79.7%) underwent genetic testing, corresponding to 4.0% (95% CI: 3.0–5.0%) of all adults in the cohort. Data on the type of genetic testing were available for 44 of the 59 tested patients: 35 were genetically tested as screening (with panels), 6 were targeted, 2 had incidental findings, and 1 underwent confirmatory testing of a variant identified in tumor tissue.

### Results of genetic testing

**Fig. 2:**
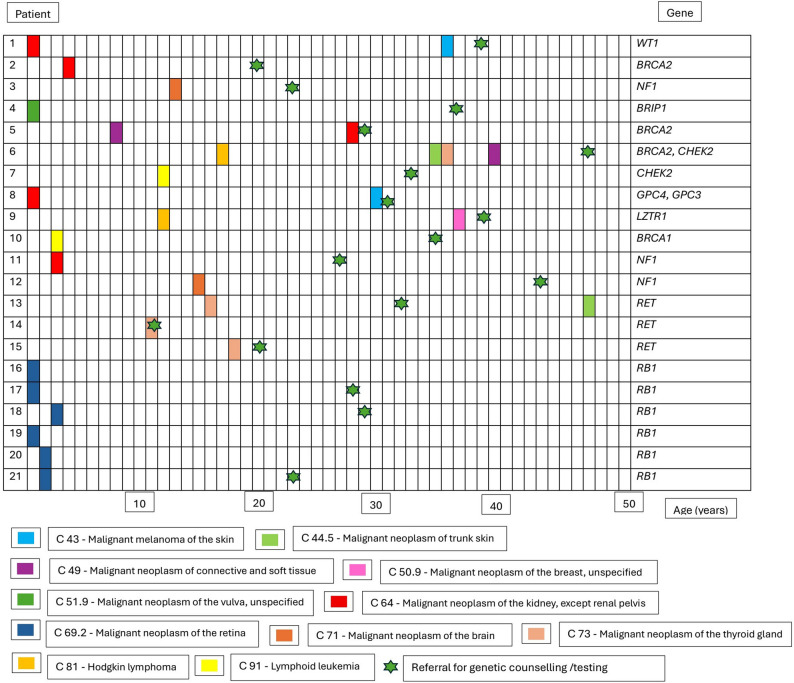
Presentation of carriers of the P/LPV, their diagnoses, and genetic testing in relation to the patient’s age at individual events, and the gene with the identified variant. Legend: C69.2 (Malignant neoplasm of the retina), C64 (Malignant neoplasm of the kidney, except renal pelvis), C73 (Malignant neoplasm of the thyroid gland), C91 (Lymphoid leukemia), C71 (Malignant neoplasm of the brain), C51.9 (Malignant neoplasm of the vulva, unspecified), C49 (Malignant neoplasm of connective and soft tissue), C81 (Hodgkin lymphoma), C44.5 (Malignant neoplasm of trunk skin), C43 (Malignant melanoma of the skin), C50.9 (Malignant neoplasm of the breast, unspecified)

Among tested patients, 21 (35.6%) were diagnosed with germline P/LPVs in a tumor predisposition syndrome gene, 38 (64.4%) tested negative, and 15 of the referred patients did not opt for testing. The mean age at testing was 34.7 ± 1.4 years (median age 35 years), with carriers of P/LPV tested at significantly lower age than non-carriers (30.4 vs. 37.0 years, *p* = 0.03). The mean interval between childhood cancer diagnosis and genetic testing was 25.7 ± 1.4 years. Figure 2 presents the characteristics of the sample of P/LPV carriers.

P/LPV in *RB1* gene were the most frequently identified (28.6%), followed by other genes as summarized in Table 1. In 61.9% of P/LPV carriers, the identified variant could explain the occurrence of primary childhood/adolescent cancer, and in 95.2% of carriers it could explain the occurrence of adult onset cancer/HCPS. Table 1 summarize the identified L/LPV, mutation types and possible association with childhood/adolescent and adult cancers.

**Table 1:** P/LPV identified in hereditary cancer predisposition genes among tested patients

Gene	*N* (%)	*P*/LPVs detected in patients	Associated with childhood and/or adolescent cancer	Associated with adult onset cancer
*P*/LPV likely causative of the childhood cancer				
*RB1* ^*i*^	6; 28.6%	*RB1* in exon 4, duplication of one nucleotide, frameshift, stop codon	YES	YES
		*RB1* in exon 19, deletion of one nucleotide		
		c.607 + 1G		
*NF1*	3; 14.3%	c.1748 A > G p.(Lys583Arg)	YES	YES
		c.4573delCp.(Leu1525Trp*28)		
		c.730G > A p.(Glu244Lys)		
*RET* ^*ii*^	3; 14.3%	L790F	YES	YES
		c.1853G > T p.(Cys618Phe)		
		C634Y		
entire *GPC4* gene and part of *GPC3* gene^ii^	1; 4.8%	duplication of Xq26.2	YES	NO
*WT1*	1; 4.8%	c.661 + 1G > C p.	YES	YES
Additional (incidental) P/LPV, unlikely related to childhood cancer				
*BRCA2*	2; 9.5%	c.3975_3978dup (p.Ala1327Cysfs*4)	NO	YES
*BRCA1*	1; 4.8%	c.181T > G p.(Cys61Gly)	NO	YES
*BRCA2* + *CHEK2*	1; 4.8%	c.6405_6409del p.(Asn2135Lysfs*3) exon 9–10 deletion c.(908+1_909-1) (1095+1_1096-1)del p.	NO	YES
*BRIP1*	1; 4.8%	c.2992_2995del p.(Lys998Glufs*60)	NO	YES
*CHEK2*	1; 4.8%	c.1100delC p.(Thr367Metfs*15)	NO	YES
*LZTR1*	1; 4.8%	c.2062 C > T p.(Arg688Cys)	YES^iii^	YES

Legend: ^i^ For three patients we do not have detailed information on the type of P/LPV; ^ii^ We do not have more detailed information on type of P/LPV; ^iii^ Pathogenic variants in *LZTR1* are relatively frequent in population databases such as gnomAD, while *LZTR1*-related schwannomatosis remains a rare condition, indicating low penetrance. Therefore, in the absence of a suggestive personal or family history, such findings should be interpreted with caution and are best considered incidental [9]; P/LPV – Pathogenic/Likely pathogenic variant; HPS – Hereditary predisposition syndrome. Reference sequences used: *BRCA2* (NM_000059.), *BRCA1* (NM_007294.3), *BRIP1* (NM_032043.3), *CHEK2* (NM_007194.4), *NF1* (NM_000267.3, *WT1* (NM_024426.6)

### Diagnosis of SPCs

Six patients (28.6%) with P/LPV developed a SPC, most commonly basal cell carcinoma and malignant melanoma, two of them developed multiple malignancies Among six patients with P/LPV and SPC, two had SPC possible associated with the HCPS (Table 2). Compared to non-carriers, the mean number of all malignancies (primary cancers and SPC) per patient was numerically lower in the positive group (1.4 vs. 2.0).

**Table 2:** Association between SPCs and the corresponding P/LPV in our 6 patients

Gene/Hereditary Syndrome	Primary cancer diagnosis in childhood and/or adolescence (ICD-10), age at the diagnosis	Link between HPCS and primary cancer in childhood and/or adolescence	SPC diagnosis (ICD-10), age at the diagnosis	Link between HPCS syndrome and SPC
*WT1* / Wilms tumor syndrome	C64 (Malignant neoplasm of kidney, except renal pelvis), age 1	YES	C43.7 (Malignant melanoma of lower limb), age 36	NO
*BRCA2*/HBOC	C49.0 (Malignant neoplasm of connective and soft tissue of head, face, and neck), age 8	NO	C64 (Malignant neoplasm of kidney, except renal pelvis), age 28	NO
*BRCA2* + *CHEK2*/ HBOC	C81.1 (Hodgkin lymphoma, classical, nodular sclerosis type), age 17	NO	C44.5 (Malignant neoplasm of skin of trunk), age 35; C73 (Malignant neoplasm of thyroid gland), age 36; C49.2 (Malignant neoplasm of connective and soft tissue of lower limb, including hip), age 40	C49.2 (Malignant neoplasm of connective and soft tissue of lower limb, including hip); possible association with increased risk in *CHEK2* mutation [10]
Entire *GPC4* gene and part of *GPC3* gene / Simpson-Golabi- Behmel syndrome	C64 (Malignant neoplasm of kidney, except renal pelvis), age 1	YES	C43.5 (Malignant melanoma of trunk), age 30	NO
*LZTR1/* schwannomatosis	C81.0 (Hodgkin lymphoma, nodular lymphocyte predominant type), age 12	NO	C50.9 (Malignant neoplasm of breast, unspecified), age 37	Conditionally YES [11]
*RET*/MEN2	C73 (Malignant neoplasm of thyroid gland), age 16	YES	C44.5 (Malignant neoplasm of skin of trunk), age 48	NO

Legend: HBOC - Hereditary breast and ovarian cancer syndrome; MEN2 – Multiple endocrine neoplasia, type 2

The most frequent primary cancers among carriers of P/LPVs were retinoblastoma (28.6%), Wilms’ tumor (19.0%), and thyroid cancer (14.3%). Mean age at first malignancy was 6.8 years in the positive group and 8.7 years in the negative group, with no significant difference (*p* = 0.21). Mean age at the time of SPC was slightly higher among carriers of P/LPV (35.7 vs. 30.6 years), but this difference was not statistically significant (*p* = 0.38).

### Family history and genetic testing of relatives

Family history was available for 6 (28.6%) of 21 carriers of P/LPV. Among these six patients, half reported a history of cancer in relatives. Genetic testing was documented for 35 relatives of 8 carriers of P/LPV (mean 3.4 additional individuals tested per family). Therefore, cascade genetic testing was performed in 38.1% (8 of 21) of all positive families and 21 of 35 (60%) relatives were positive at testing.

Thirteen (37.1%) of 35 tested relatives developed malignancy, most frequently breast and thyroid cancer. Among these relatives the interval from cancer diagnosis to genetic testing ranged from 1 to 21 years, with a mean of 5.4 ± 2.0 years and a median of 2 years (IQR 1–6 years). 47.7% (10/21) of carriers of the P/LPV developed malignancy, and in as many as 9 (90%) of these cases, the cancer could be linked to the identified hereditary cancer predisposition. Five cases involved adult-onset cancer genes (*BRCA1*, *CHEK2*, *ATM*, *BRCA2*), while four were *RET* P/LPVs, which can be associated with childhood as well as adult tumors. All hereditary-related cancers in relatives occurred in adulthood (ages 19–72).

## Discussion

This study provides the first population-based analysis of genetic testing for hereditary cancer predisposition syndromes among adult survivors of childhood and adolescent cancer in Slovenia as growing scientific evidence highlights the critical role of genetic testing in pediatric oncology. We found that only a small proportion of survivors were referred for (5,0%) or underwent (4,0%) genetic testing; however, approximately one third of those tested (35.6%) had a positive genetic result. The most frequently detected P/LPV were in *RB1*, *NF1*, *RET* and *BRCA2* genes. Among 21 carriers of the P/LPV, occurrence of cancer during childhood/adolescence could explained by a HPCS in 13 (61.9%) patients. Most (95.2%) identified syndromes were also linked to adult-onset cancers. Cascade testing revealed a high proportion of affected relatives (60%), most of whom developed malignancy consistent with identified HCPS.

### Genetic testing uptake and testing results

Direct comparison of our results with existing literature is limited, as only a few studies have examined genetic testing in adult survivors of childhood or adolescent cancer. Nonetheless, it is well established that only a minority of cancer patients, regardless of age, are referred for genetic testing. This finding is consistent with recent reports, such as Kurian et al. [12], showing generally low uptake of genetic testing among cancer patients. Recent studies report that HCPSs account for 7–15% of pediatric cancers [13]. In a recent study (National Cancer Institute–Children’s Oncology Group Pediatric MATCH Trial), Scollon et al. analysed tumor and blood DNA from patients aged 1–21 years with treatment-refractory cancers, non-Hodgkin lymphomas, or histiocytic disorders. P/LPVs were identified in 6.3% (73 of 1,167) of germline reports across 21 genes previously linked to pediatric and/or adult cancers. The most frequently mutated genes with concurrent germline findings were *NF1* and *TP53*, whereas genes such as *ALK* and *PTEN* were less commonly altered or lacked germline variants. The simultaneous detection of pathogenic variants in genes primarily associated with adult-onset cancers highlights the frequent identification of incidental findings in children and adolescents and underscores the need for careful germline interpretation following tumor genomic testing [14].

In our cohort, roughly one third of tested individuals carried a PV/LPV, most frequently in *RB1*, along with several other cancer predisposition genes. Notably, our study focused on adult survivors of childhood and adolescent cancer (aged 25 years or older, born between 1970 and 2000), many of whom were diagnosed at a time when genetic testing was less accessible [15]. This explains the relatively high average ages at referral for testing in our sample and the longer time interval between first cancer diagnosis and referral for or actual genetic testing (mean 25.7 ± 1.4 years). It is worth noting that during data review, we also identified several patients who developed cancer in early childhood but died soon after diagnosis. On the other hand, some patients in the cohort declined genetic testing despite being referred multiple times (we identified six such cases). Other differences in findings compared with previous studies may be due to study design (our study was population-based), cancer types, testing strategies, and population characteristics.

### Diagnosis of second primary cancers in survivors

Childhood cancer survivors with germline P/LPV in cancer predisposing genes are believed to have an increased risk of SPC later in life. Wang et al. at St. Jude Hospital found that survivors with PV/LPV who had not received radiotherapy had nearly six fold higher odds of developing SPC than those without such variants (OR = 5.6; 95% CI: 2.6–12) [16, 17]. In our cohort, however, the average number of cancer diagnoses per patient differed significantly between positive and negative individuals (1.4 ± 0.2 vs. 2.0 ± 0.2, *p* = 0.03), in negative ones being even higher. However, this result should be interpreted with caution, as the small size of the positive group (carriers of the P/LPV) may limit the robustness of the finding. Patients with germline P/LPV generally develop associated cancers at younger ages than negative patients [18]. The average age at diagnosis of childhood cancer is around 7.3 years [19, 20]. In our sample, P/LPV carriers were slightly younger at first diagnosis of malignant disease than non-carriers (6.8 ± 1.4 vs. 8.7 ± 1.0 years), although this difference was not statistically significant (*p* = 0.21). A significant difference was, however, found in age at referral for genetic testing (*p* = 0.03), with carriers being referred earlier, likely reflecting stronger clinical suspicion of hereditary predisposition.

Among six positive patients who later developed SPC, only two cases could be possibly linked to identified HCPSs. These findings suggest that most SPCs in our sample were likely due to other factors (primary cancer treatment, environmental, or lifestyle) rather than heredity alone. Four carriers of P/LPV harboured *BRCA1* or *BRCA2* gene variants (twice in *BRCA2*, once in *BRCA2 + CHEK2*, and once in *BRCA1* gene). Although heterozygous P/LPV in *BRCA1* and *BRCA2* genes are rarely linked to pediatric cancers, such findings justify early surveillance in adulthood, given their known association with HCPSs and SPCs.

### Family history and cascade genetic testing

Family history was available for 6 of 21 carriers of P/LPV with half reporting a history of cancer in relatives. We also analysed 35 relatives of eight carriers of P/LPV. In Slovenia, cascade testing of relatives is part of the public healthcare system, allowing all blood relatives of a proband with a known PV/LPV to undergo testing without additional costs for the patient [21]. Testing is voluntary and depends on the proband’s communication with relatives, since legal restrictions prevent healthcare professionals from contacting relatives directly. Data from the literature show that such family-based cascade testing leads to testing in only about 36% of relatives, leaving the rest uninformed about their potential risks and preventive options [22].

In our study, 60% (21/35) of relatives tested positive. Ten of these relatives developed malignant disease, and one developed SPC. In 90% (9/10) of these patients, cancer could be linked to the identified HCPS. All associated tumors occurred in adulthood (ages 19–72). The time between first cancer diagnosis and genetic testing among relatives (mean 5.4 years) was much shorter than among 21 carriers of the P/LPV from our cohort (25.7 years), suggesting that earlier testing and surveillance might allow prevention or early detection of malignancies in positive relatives. Appropriate preventive measures and regular monitoring could prevent or enable early detection of cancer, when treatment is more effective with less side sequelae. Studies from St. Jude Children’s Research Hospital have shown that early surveillance frequently detects asymptomatic tumors, about half of which can be surgically removed, avoiding toxic therapies. At St. Jude, monitoring typically begins several months to years after detection of genetic predisposition or once treatment of the primary tumor is completed, to avoid overlooking SPC [23]. It is also important to emphasize that even individuals with negative results should receive genetic counselling to understand their baseline population risk and the importance of routine screening [21].

### Limitations of our study

The main limitation of our study is its relatively small sample size of tested individuals, particularly for detailed analysis of patients harbouring PV/LPV in cancer predisposing genes. However, this is partly due to Slovenia’s small population size and the limited number of referrals. False negatives are also possible due to targeted testing methods that overlook mutations outside known familial variants. Although not strictly considered a limitation, we must also acknowledge that not all cancer-associated genes are yet known, and some mutation-negative patients may still harbour undiscovered predispositions. As genetic testing becomes increasingly available during childhood, around the time of diagnosis, this issue is expected to diminish and predominantly reflects evolving clinical practice rather than a flaw in the study.

Despite its limitations, this study supports the integration of genetic testing into standard care for childhood and adolescent cancer patients. When universal testing is not feasible, targeted testing based on clinical suspicion should be strongly considered, particularly for syndromes associated with adult-onset malignancies. Accordingly, The European working group of experts in the field of genetics (SIOPE Host Genome Working Group) has issued recommendations for genetic testing in pediatric cancer patients [24]. They advise that all such patients undergo clinical screening for HCPSs and targeted genetic testing based on clinical indications. These recommendations have also been implemented in recent years at the Clinical Department of Pediatric Hematology and IOL. Early identification of hereditary cancer predisposition enables personalized surveillance, timely detection of subsequent cancers, and risk reduction for both survivors and their relatives.

## Conclusions

Our study confirmed that only a small proportion of adult childhood or adolescence cancer survivors undergo genetic testing for hereditary cancer predispositions. Among those tested, several clinically relevant P/LPV were identified, most commonly in *RB1*, *NF1*, *RET* and *BRCA2* genes. In many patients with positive genetic test, the occurrence of childhood cancer could be explained by an underlying HCPS, some of which are also linked to cancers in adulthood. Early genetic testing and systematic follow-up of patients and their relatives could enable earlier detection or even prevention of new malignancies through targeted surveillance and timely interventions.

## Data Availability

The datasets generated and/or analyzed during the current study are not publicly available due to institutional restrictions but are available from the corresponding author on reasonable request.

## Abbreviations

AML: Acute myeloid leukemia
*ATM*: Ataxia-telangiectasia mutated (gene)
*BRCA1*: BRCA1 DNA repair-associated protein (gene)
*BRCA2*: BRCA2 DNA repair-associated protein (gene)
*BRIP1*: BRCA1-interacting protein (gene)
CI: Confidence interval
CIGM: Clinical Institute of Genomic Medicine
CPG: Cancer predisposition gene
CPS: Cancer predisposition syndrome
DOCG: Department of Oncological Clinical Genetics
*GPC3*: Glypican 3 (gene)
*GPC4*: Glypican 4 (gene)
HBOC: Hereditary breast and ovarian cancer
ICD-10: International Statistical Classification of Diseases and Related Health Problems, 10th Revision
IOL: Institute of Oncology Ljubljana
IQR: Interquartile range
LTFUC: Long-term Follow-Up Clinic
LPV: Likely pathogenic variant
*LZTR1*: Leucine zipper like post translational regulator 1 (gene)
MEN2: Multiple endocrine neoplasia type 2
*NF1*: Neurofibromin 1 (gene)
PV: Pathogenic variant
*RB1*: RB transcriptional corepressor 1 (gene)
*RET*: RET proto-oncogene (gene)
SCR: Slovenian Cancer Registry
SPC: Second primary cancer
WHO: World Health Organization
*WT1*: WT1 transcription factor (gene)
